# CBCTs in a Swiss university dental clinic: a retrospective evaluation over 5 years with emphasis on radiation protection criteria

**DOI:** 10.1007/s00784-023-05184-y

**Published:** 2023-07-31

**Authors:** Samuel Klingler, Philippe Biel, Moses Tschanz, Ralf Schulze

**Affiliations:** 1 Department of Oral Surgery and Stomatology and Oral Diagnostics, School of Dental Medicine, University of Bern, Freiburgstrasse 7, 3010 Bern, Switzerland; 2 Information Technology (IT) Group, School of Dental Medicine, University of Bern, Freiburgstrasse 7, 3010 Bern, Switzerland; 3 Division of Oral Diagnostic Sciences, Department of Oral Surgery and Stomatology and Oral Diagnostics, School of Dental Medicine, University of Bern, Freiburgstrasse 7, 3010 Bern, Switzerland

**Keywords:** Radiation protection, Radiation dosage, Cone-beam computed tomography, Pediatric radiography, Dose optimization

## Abstract

**Objectives:**

To retrospectively evaluate all cone-beam computed tomography (CBCT) scans acquired from 2017 to 2022 in a Swiss university dental clinic with particular emphasis on radiation protection aspects.

**Material and methods:**

Radiological databases at the dental clinic of the University of Bern, Switzerland, were explored using a self-developed search algorithm. Data of all acquired CBCT from 01.01.2017 to 27.06.2022 were screened. Exposure parameters (exposure time, exposure angle, milliampere (mA), kilovoltage (kV), field of view (FOV) size), dose area product (DAP), age, and sex of the patient were recorded anonymously. The collected data were analyzed mainly descriptively. Correlations measured the statistical relationships between the variables.

**Results:**

A total of 10,348 CBCT datasets were analyzed. Patient age ranged from 5 to 96 years (mean: 49.4 years, SD: 21.6 years). The number of CBCTs in patients under 25 years was around 20% each year. In total, 10,313 (99.7%) CBCTs were acquired in small to medium FOV (FOV up to 10 cm of height), and 35 (0.3%) in large FOV (height > 10 cm). DAPs of small FOVs were 518.3 ± 233.2 mGycm^2^ (mean ± SD), of medium FOV 1233 ± 502.2 mGycm^2^, and of large FOV 2189 ± 368.7 mGycm^2^. DAP (*ρ* = 0.4048, *p* < 0.0001) and kV (*ρ* = 0.0210, *p* = 0.0327) correlated positively with age. Reduced scan angle correlated with young age (*r*_*pb*_ 0.2729, *p* < 0.001). mA did not correlate with age (*p* = 0.3685).

**Conclusions:**

This study demonstrates that certain well-known radiation protection aspects as the reduction of FOV, mA, kV, and scan angle were only partly considered.

**Clinical relevance:**

Known radiation protection aspects, especially in young patients, should be fully applied in regular clinical practice.

## Introduction

Dental radiographs are high-frequency radiographic procedures, with approximately 13% of all diagnostic radiological examinations globally being performed in dentistry [1]. Interestingly, the annual frequency of dental radiographs is estimated to be 74 examinations per 1000 population globally, while it amounts to approximately 275 examinations per 1000 population in level 1 countries [1]. An important factor in dentistry is that dentists in many countries undertake X-ray procedures for patients based on their own clinical assessment, i.e., they justify and acquire the radiographs themselves. This process is generally termed “self-referral” [2] which “leads to potential weaknesses in the justification process due to a lack of objectiveness, possibly also driven by economic considerations” [3].

Maxillofacial cone-beam computed tomography (CBCT) has been available for more than 20 years now [4, 5]. It represents the three-dimensional (3D) radiographic imaging technique in dentistry, and its advantages and disadvantages are well understood. We can currently observe an increasing trend in acquiring CBCT images due to the emerging digital workflow in dentistry, the steadily decreasing radiation dose of CBCTs, and the availability of CBCT devices [6, 7]. In 2019, there were about 700 installed CBCT devices in Switzerland (1.2 units per 10,000 inhabitants), with an ongoing increasing trend [7]. As the radiation dose involved with CBCT scans is considerably higher than the one from typical two-dimensional (2D) dental radiographic imaging [8], many guidelines for the safe use of CBCTs have been published (for an overview, see e.g., Horner et al. [9]). Furthermore, in a safety report on radiation protection in dental radiology published by the International Atomic Energy Agency (IAEA) in 2022, the radiation protection in CBCT use is discussed in detail [3].

Technical as well as biological parameters influence the patient radiation dose of CBCTs. While the technical parameters, like the exposure factors, can often be adjusted, the biological parameters are given. Thus, age has a significant impact. Children are susceptible to the carcinogenic effects of ionizing radiation due to cell growth, organ development, and longer life expectancy [8, 10]. Although the risk of craniofacial imaging to the individual is generally small, there is a lack of pediatric studies in this area [10].

Given the higher radiation dose involved with CBCT and the available guidance for its safe use, it appears interesting to have a closer look at the routine CBCT imaging practice in a university dental clinic in a Level 1 country. This retrospective investigation aims to statistically evaluate all CBCT scans conducted from 2017 to 2022 at the Division of Oral Diagnostic Sciences at the dental clinic (School of Dental Medicine) of the University of Bern, Switzerland. Emphasis is on factors relating to radiation protection aspects, such as patient age, exposure factors (field of view (FOV) size, milliampere (mA), kilovoltage (kV), scan angle), and the dose area product (DAP), with a particular focus on children, juveniles, and young adults.

## Materials and methods

Using a script developed by MT, the databases of the Division of Oral Diagnostic Sciences at the dental clinic of the University of Bern, Switzerland, were searched over the time period 01.01.2017 to 27.06.2022. The CBCTs were acquired with the 3D Accuitomo 170 or the Veraview X800 (both J. Morita Corp., Kyoto, Japan). All data from CBCT images acquired over this period were screened. Exposure parameters (exposure time, exposure angle, milliampere (mA), kilovoltage (kV), and field of view (FOV) size), as well as the dose area product (DAP), age and sex of the patients, were recorded in an Excel-Sheet (Excel 2016, Microsoft Corporation, Redmond, Washington, USA) in a fully anonymous fashion. For this retrospective study, only the previously mentioned parameters were collected anonymously. No CBCT images were viewed, and no medical records were consulted. The justification of the CBCT images is not noted in the database and, therefore, could not be collected from the database. The study follows STROBE recommendations for observational studies [11]. Since no patient-related information was retrieved, according to the Federal Act on Research Involving Human Beings of the Swiss Federal Law (Human Research Act, HRA, Switzerland), this quality control study does not require ethical clearance.

### Search algorithm

For all radiology data in the Division of Oral Diagnostic Sciences at the dental clinic of the University of Bern, two databases of i-Dixel (i-Dixel Software, J. Morita Corp., Kyoto, Japan) exist. Two databases were established due to a system migration needed to introduce a digital medical history from 14.09.2020 onwards. Consequently, one i-Dixel database contained all data from 01.07.2004 to 13.09.2020, and the other i-Dixel database contained all data from 14.09.2020 to 27.06.2022. Two different scripts based on the Structured Query Language (SQL) and Python 3.10 were written by MT to search the databases. Generally, all parameters of interest (exposure time, exposure angle, mA, kV, DAP, FOV size, age, and sex) were extracted with an SQL script from the tables patient, series_info (Excel was used for the splitting attendant_data field), and photo_info. For the older database (01.07.2004–13.09.2020), an additional Python script was used to extract the Acquisition Mode and CtTaskId from the CT Directory (Constants1100.xml and CtStatus.csv) because the Acquisition Mode was missing in this database. Subsequently, all data up to 31.12.2016 were excluded to evaluate at least 5 years.

### Data evaluation

**Fig. 1:**
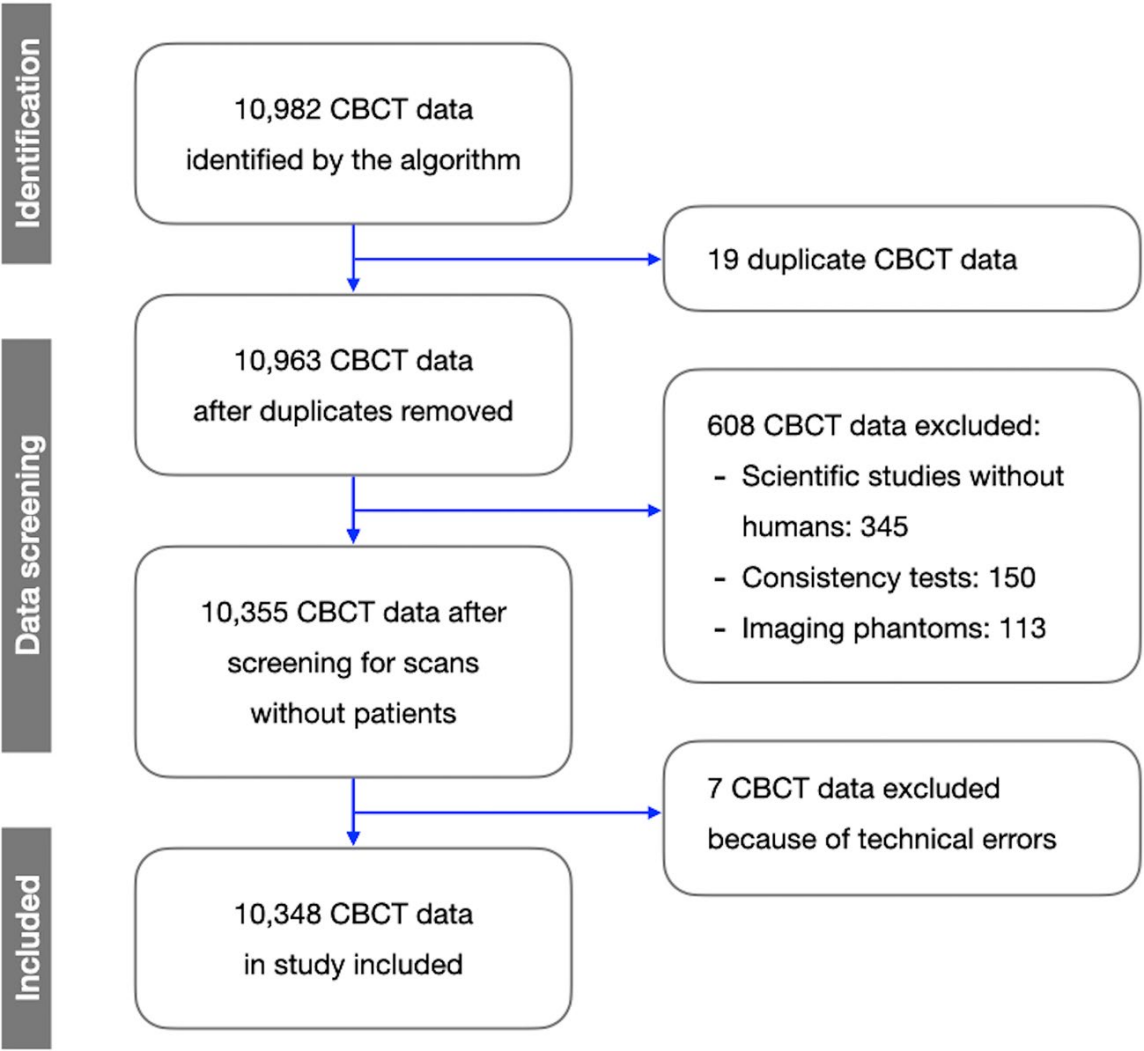
Flow chart of the data collection

From the period 01.01.2017 to 27.06.2022, 10,982 CBCT data recordings were extracted by the algorithm. Nineteen (*n* = 19) datasets were excluded due to duplicates according to their identical, uniquely assignable CT number. Six hundred eight (*n* = 608) CBCT data were excluded because the scans were made without human patients (scientific studies without humans, consistency tests, and imaging phantoms). These datasets were found due to specific data labeling, a standardized procedure in the Division of Oral Diagnostic Sciences. Seven (*n* = 7) CBCT datasets were excluded because of an irregularity in the exposure time in the context of technical errors in image acquisition, e.g., power interruption. All excluded data were manually checked against the scan parameters of the CBCT databases in the CBCT viewing software (i-Dixel Software, J. Morita Corp., Kyoto, Japan) by two authors (SK, PB). The data collection procedure is illustrated in a flow chart in Fig. 1.

Finally, a list of 10,348 CBCT data resulted. These data were manually compared with an existing list for internal statistics for all parameters extracted by two authors (SK, PB). Missing values were inserted manually. The extracted data were further manually analyzed with the scan parameters of the CBCT databases in the CBCT viewing software (i-Dixel Software, J. Morita Corp., Kyoto, Japan). No irregularities were found for either comparison.

### Statistical analysis

Using R language and environment for statistical computing [12], the data were analyzed mainly in a descriptive fashion. The collected data were summarized using descriptive statistics and presented in box- or violin plots and tables. Statistical relationships were calculated by Spearman’s correlation or point-biserial correlation and presented in scatterplots. Group differences were analyzed with a Mann–Whitney *U* test. The library “ggplot2” was used for the graphical representation of the data [13]. All analyses were conducted considering a 95% confidence level (*p* < 0.05).

## Results

**Fig. 2:**
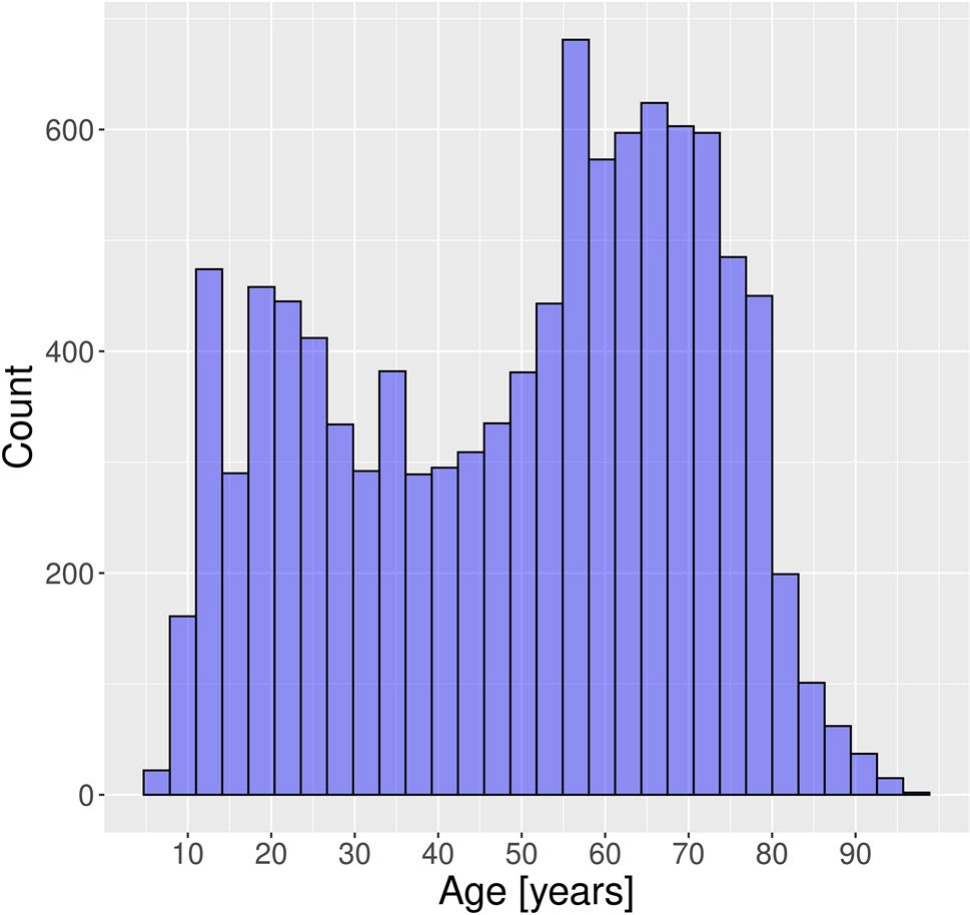
Patient age distribution of the 10,348 CBCTs

**Fig. 3:**
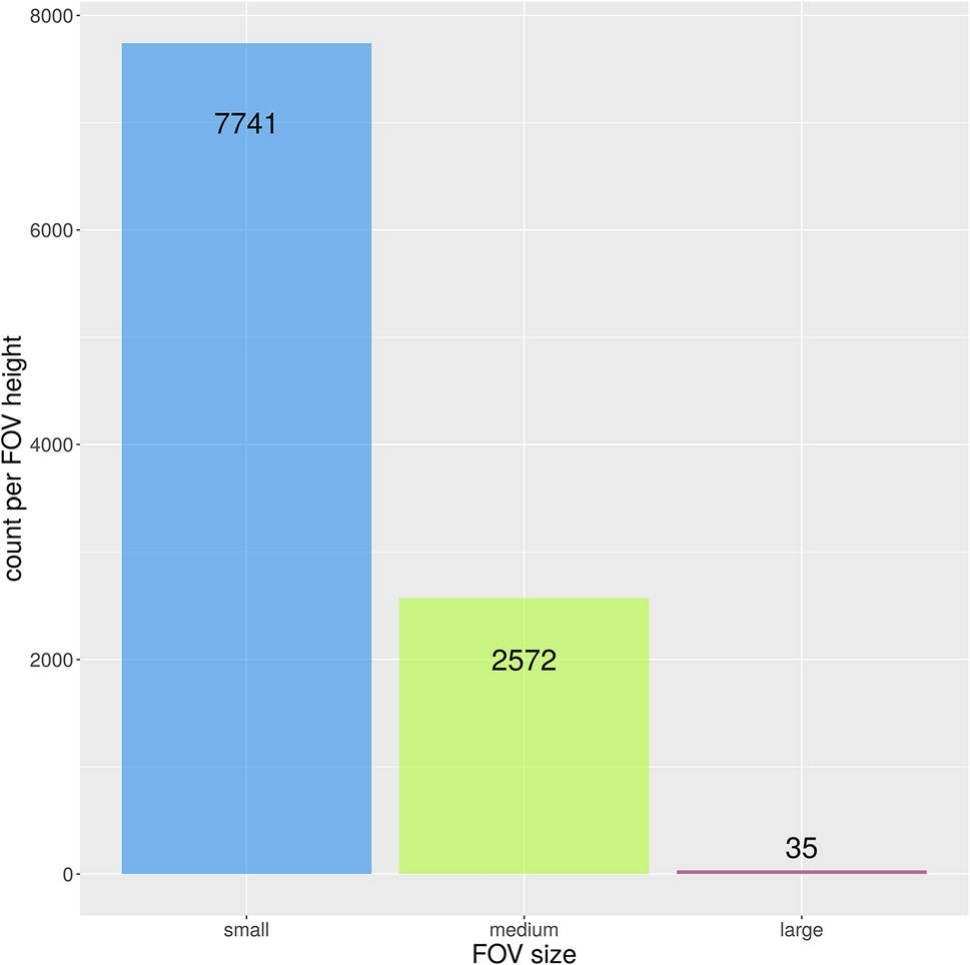
Distribution of FOV classes over the five-and-a-half-year period

**Fig. 4:**
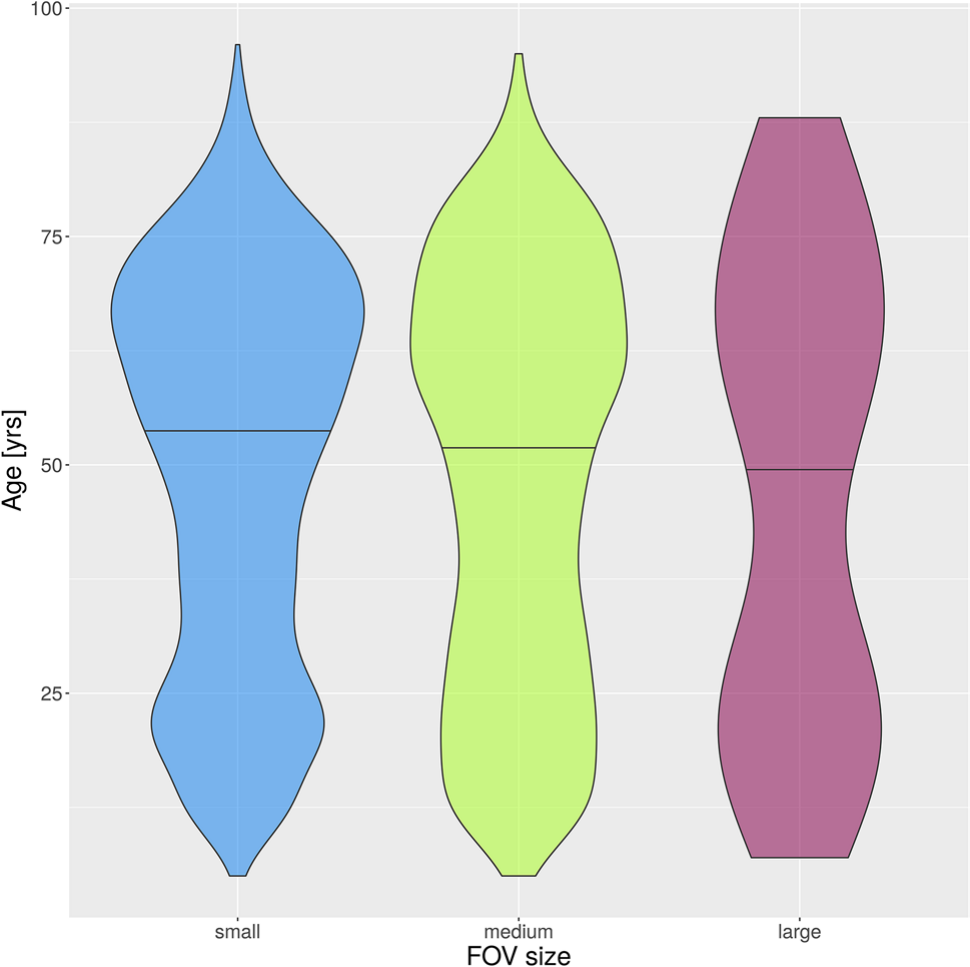
Violin plot of age distribution for the three FOV classes

A total of 10,348 CBCT images were acquired during the period from 01.01.2017 to 27.06.2022. A total of 5563 (53.8%) CBCT scans were taken in women and 4785 (46.2%) images in men. The number of CBCT scans per year was distributed as displayed in Table 1. Patient age ranged from 5 to 96 years with a mean age of 49.4 years (standard deviation SD: 21.6 years). The patient age distribution is shown in Fig. 2. Notably, the plot shows two peaks, one around the age of 20 years and the other peak between 60 and 70 years. The number of CBCTs in patients under 25 years was around 20% each year. A total of 1405 (13.6%) CBCTs have been done in patients younger than 20 years over the entire period. The distribution of the CBCTs in the juvenile age groups is shown in Table 2. When classifying the CBCTs according to FOV height (small FOV ≤ 5 cm, medium FOV > 5 cm and ≤ 10 cm, large FOV > 10 cm), the majority (10,313; 99.7%) of the CBCTs were acquired in small to medium FOV height (up to 10 cm), while very few (35; 0.3%) were acquired in large size FOV (height > 10 cm) (Fig. 3). The complete distribution of the different FOV sizes is detailed in Table 3. The median age in the small FOV size was 51 years, in medium FOV height 59 years, and in the large size FOV 57 years (Fig. 4).

**Table 1:** Number of CBCTs per year (total and per juvenile/young adults age group)

Year	Total No. of CBCTs	CBCTs in patients under 18 years	CBCTs in patients between 18 and 25 years
2017	2182	158 (7.2%)	255 (11.7%)
2018	2157	166 (7.7%)	265 (12.3%)
2019	1956	153 (7.8%)	226 (11.6%)
2020	1602	175 (10.9%)	200 (12.5%)
2021	1725	211 (12.2%)	202 (11.7%)
2022 (Jan.–Jun.)	726	84 (11.6%)	58 (8.0%)

**Table 2:** Number of CBCTs in juvenile age groups over the entire period (2012–06/2022; Total No. of CBCTs: 10,348)

Age (years)	Total No. of CBCTs in age group	Percentage of total No. of CBCTs
≤ 10	183	1.8%
10 to 15	583	5.6%
15 to 20	639	6.2%
Sum	1405	13.6%

**Table 3:** Distribution of FOV size (height × width). First row: absolute count per year, second row: percent per year

		Year						
		2017	2018	2019	2020	2021	2022 (Jan.–Jun.)	Total
FOV size (cm)	2.5 × 2.5	0 0.0%	2 0.1%	5 0.3%	3 0.2%	1 0.1%	1 0.1%	12 0.1%
	4 × 4	787 36.1%	788 36.5%	630 32.2%	518 32.3%	502 29.1%	132 18.2%	3357 32.4%
	4 × 6	219 10.0%	201 9.3%	204 10.4%	136 8.5%	164 9.5%	112 15.4%	1036 10.0%
	4 × 8	106 4.9%	84 3.9%	46 2.4%	25 1.6%	15 0.9%	16 2.2%	292 2.8%
	5 × 6	253 11.6%	269 12.5%	251 12.8%	228 14.2%	309 17.9%	107 14.7%	1417 13.7%
	5 × 8	220 10.1%	236 10.9%	174 8.9%	157 9.8%	178 10.3%	76 10.5%	1041 10.1%
	5 × 10	61 2.8%	63 2.9%	95 4.9%	93 5.8%	100 5.8%	45 6.2%	457 4.4%
	5 × 14	14 0.6%	24 1.1%	24 1.2%	18 1.1%	22 1.3%	27 3.7%	129 1.2%
	6 × 6	176 8.1%	157 7.3%	193 9.9%	187 11.7%	142 8.2%	48 6.6%	903 8.7%
	8 × 4	1 0.0%	7 0.3%	4 0.2%	2 0.1%	5 0.3%	0 0.0%	19 0.2%
	8 × 8	197 9.0%	156 7.2%	182 9.3%	75 4.7%	103 6.0%	60 8.3%	773 7.5%
	10 × 10	106 4.9%	124 5.7%	107 5.5%	115 7.2%	109 6.3%	47 6.5%	608 5.9%
	10 × 14	35 1.6%	46 2.1%	37 1.9%	44 2.7%	61 3.5%	46 6.3%	269 2.6%
	12 × 17	7 0.3%	0 0.0%	4 0.2%	1 0.1%	14 0.8%	9 1.2%	35 0.3%
Total		2182 100%	2157 100%	1956 100%	1602 100%	1725 100%	726 100%	10,348 100%

**Fig. 5:**
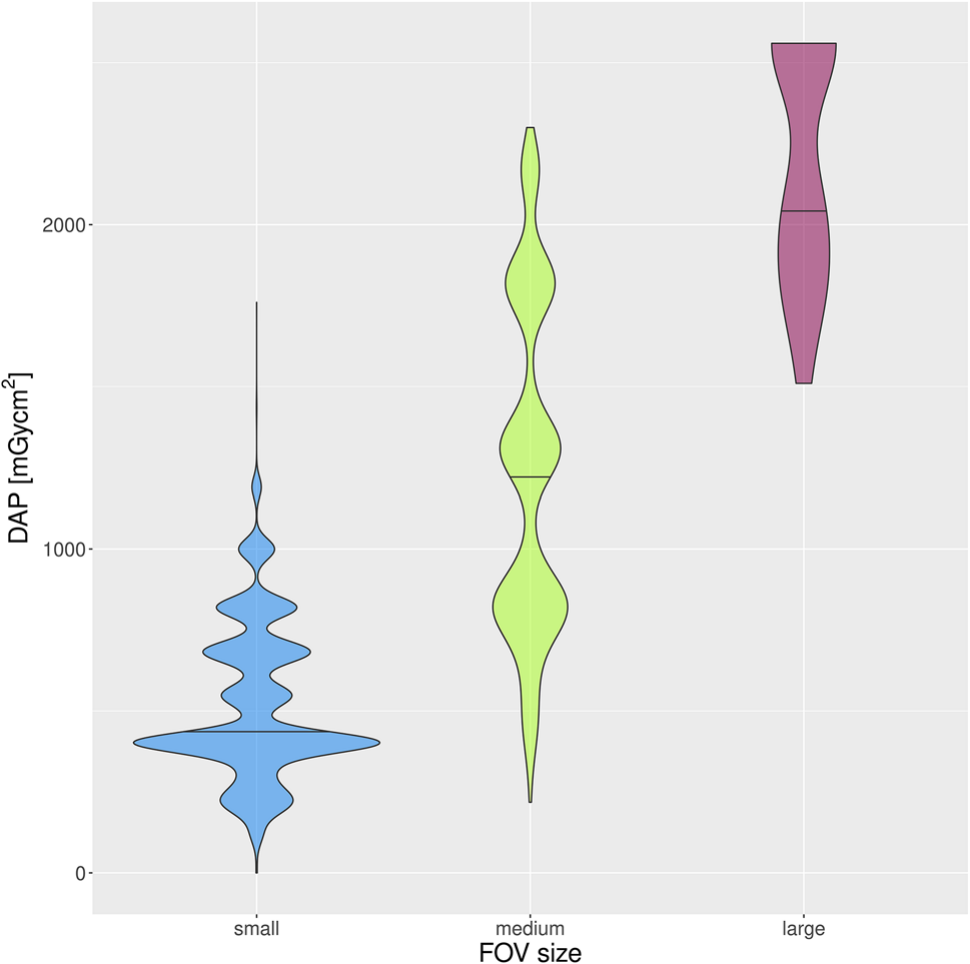
Violin plot of DAP distribution for the three FOV classes

**Fig. 6:**
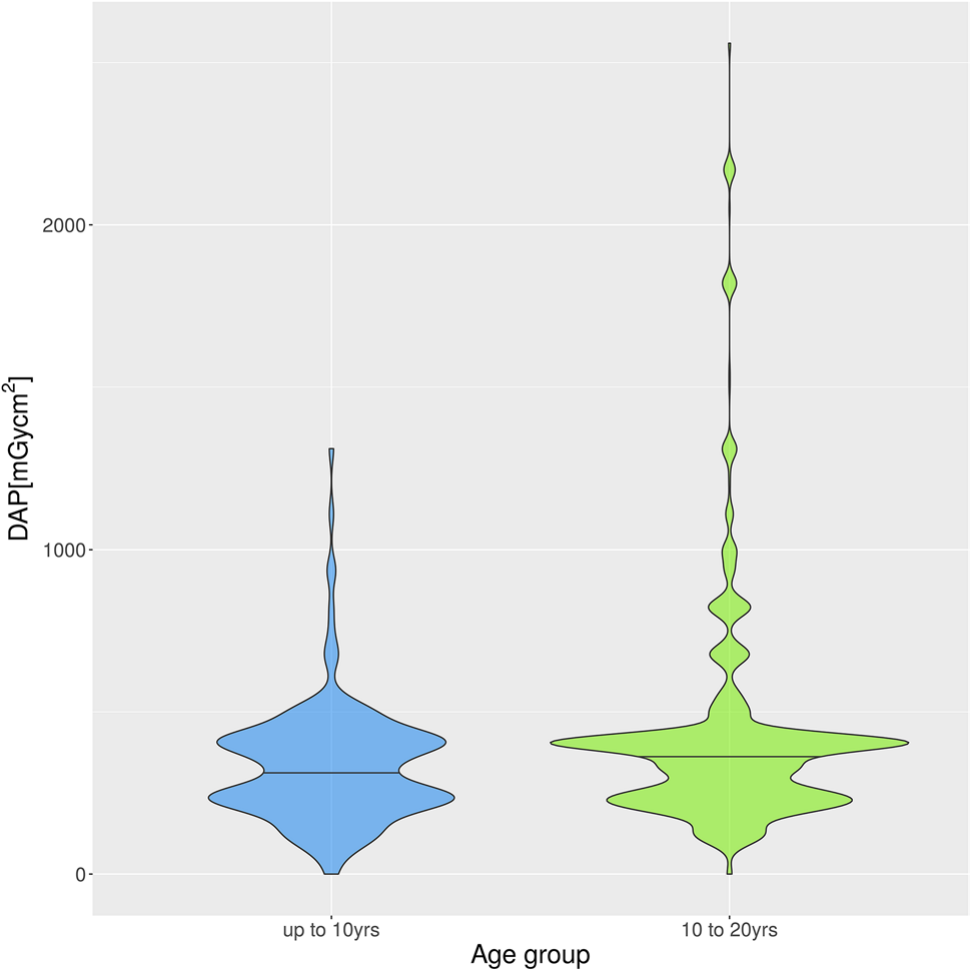
Violin plot of DAP distribution for age groups up to 10 years and 10 to 20 years

**Fig. 7:**
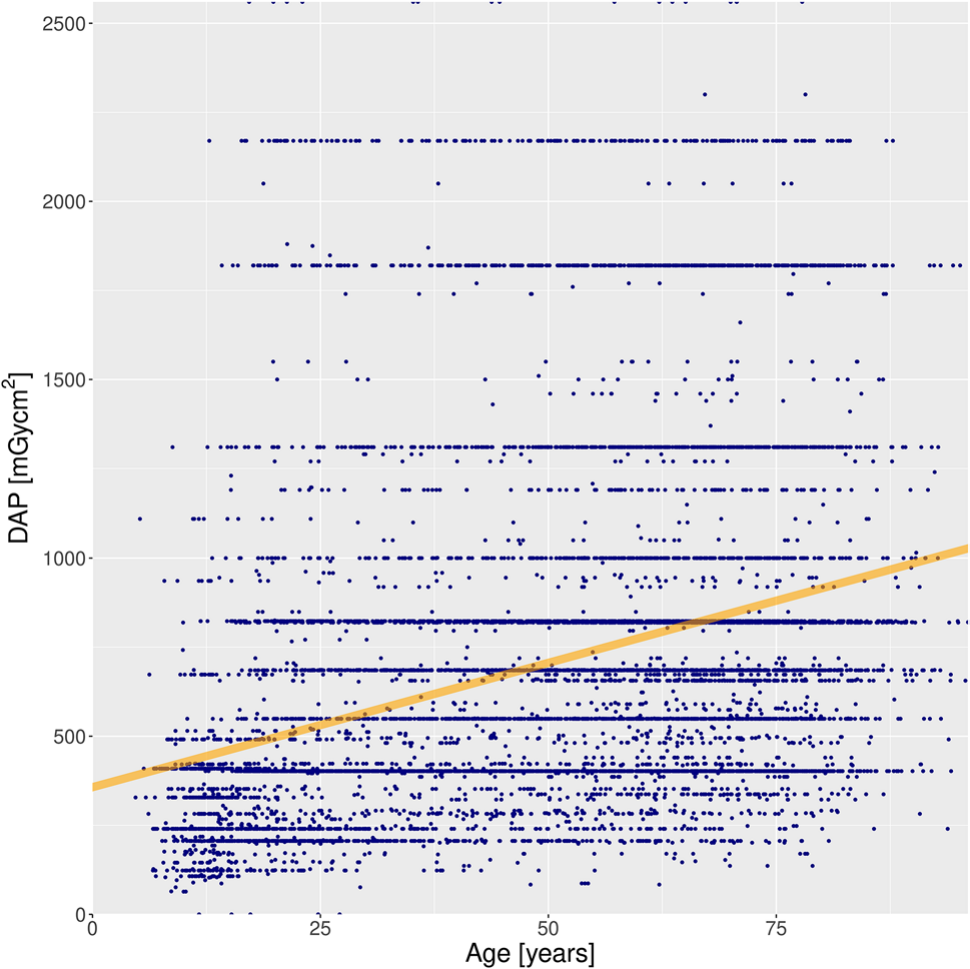
Scatterplot of DAP versus patient age. The orange line shows the fitted slightly positive linear correlation trendline (*p* < 0.0001)

DAPs of small FOVs were 518.3 ± 233.2 mGycm^2^ (mean ± SD) versus 1233 ± 502.2 mGycm^2^ for the medium and 2189 ± 368.7 mGycm^2^ for the large FOV (Fig. 5). DAPs in the age group up to 10 years were 331.1 ± 178.9 mGycm^2^ (328.0 mGycm^2^) (mean ± SD (median)) and in the age group 10 to 20 years, 424.4 ± 338.5 mGycm^2^ (402.0 mGycm^2^) (mean ± SD (median)) (Fig. 6). There was a positive correlation (*ρ* = 0.4048) between age and DAP, which was highly statistically significant (*p* < 0.0001) (Fig. 7).

**Fig. 8:**
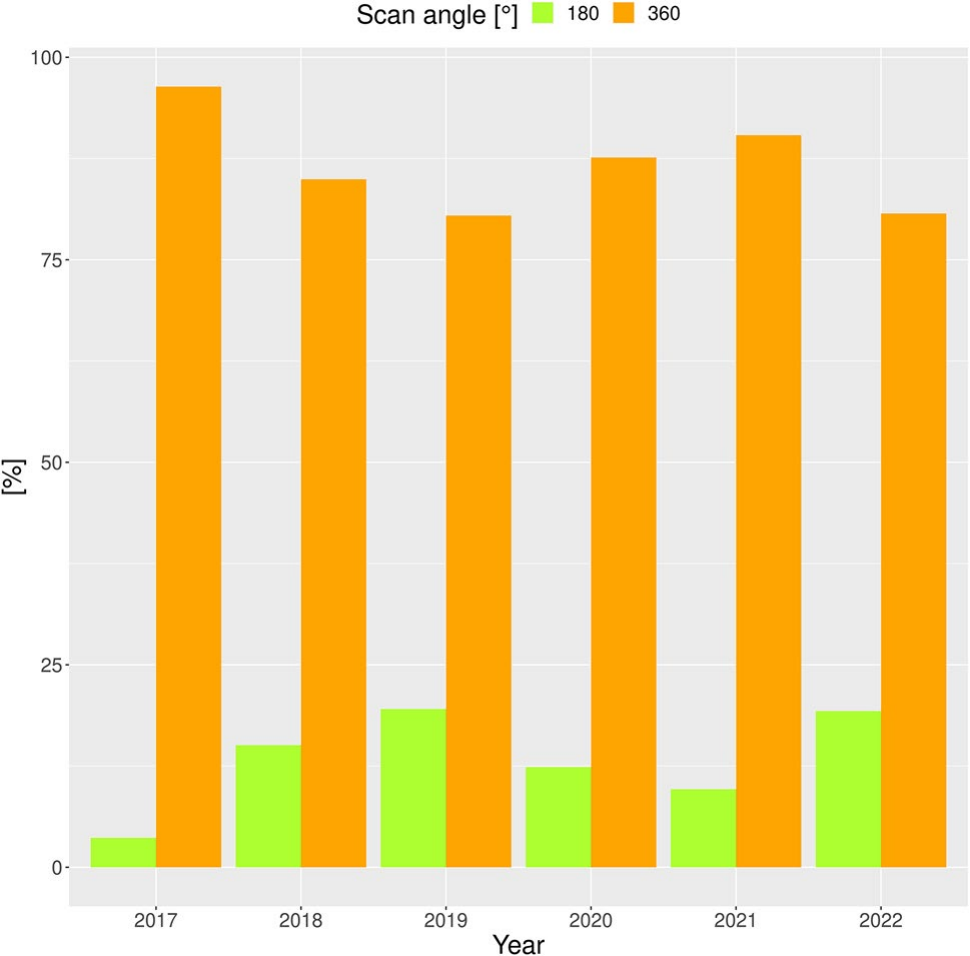
Scan angles (half = 180° versus full = 360°) applied over the five-and-a-half-year period

**Fig. 9:**
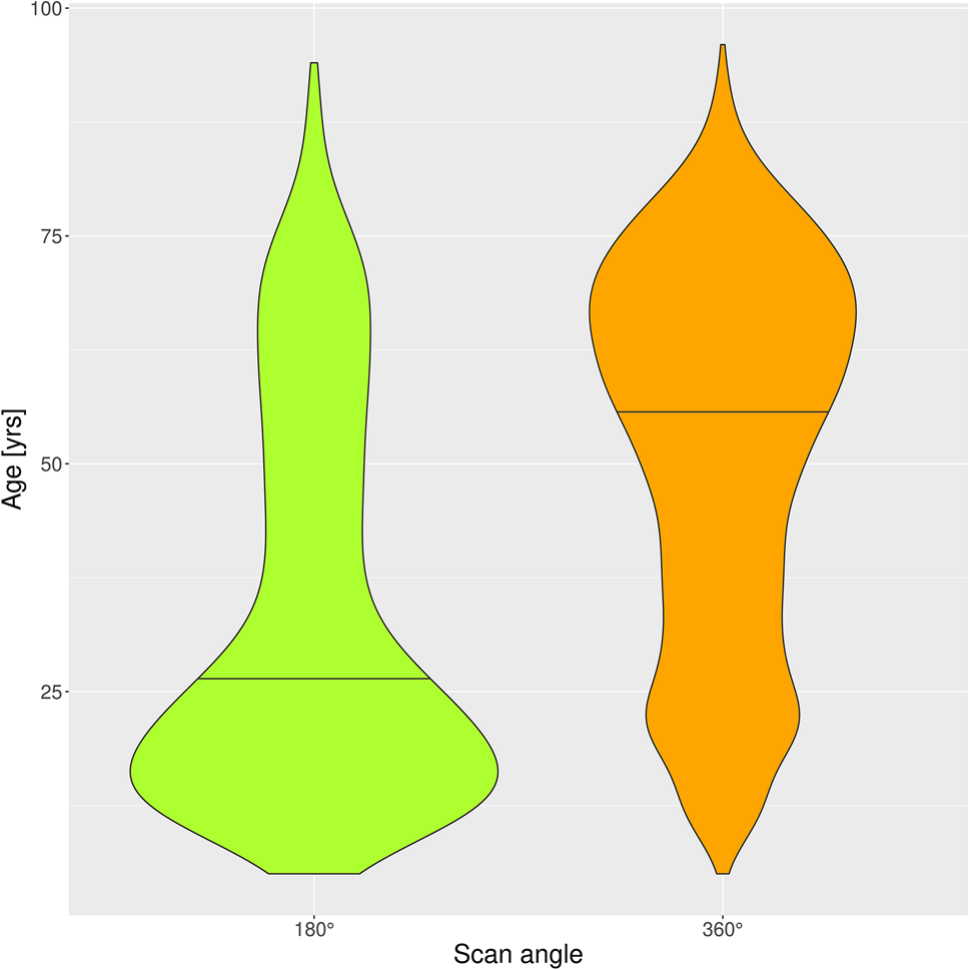
Violin plot of age distribution depending on scan angles (half = 180° versus full = 360°)

Regarding other exposure parameters, the mean value of our cohort was 4.9 ± 0.5 mA (mean ± SD) for the tube current and 89.7 ± 2.0 kV (mean ± SD) for the beam energy. mA did not significantly correlate with age (*p* = 0.3685), while kV showed a positive correlation with age (*ρ* = 0.0210, *p* = 0.0327). Reduced scan angles of 180° were applied in varying percentages over the years, with maximum values around 20% of all exposures (Fig. 8). Age distribution depending on the scan angle (180° vs. 360°) is displayed in Fig. 9. For the scan angle of 180°, patients were 33.8 years old (± 21.6 years (SD)) on average. For the full scan angle (360°), the patient’s age averaged 56.0 years (± 20.7 years (SD)). Reduced scan angle correlated with young age (point-biserial correlation *r*_*pb*_ 0.2729, *p* < 0.001). The age difference between the two scan angle groups was highly significant (Mann–Whitney *U* test, *p* < 0.0001).

## Discussion

This research retrospectively evaluated all CBCTs acquired at the dental clinic of the University of Bern, Switzerland, over a five-and-a-half-year period. A particular emphasis was placed on radiation protection by optimization of exposure parameters in children, juveniles, and young adults. In a university dental clinic environment, CBCTs are commonly acquired for external referrals as well as for internal patients. The diagnostic spectrum of such an environment is broad, and the number of CBCTs will undoubtedly be much larger than those acquired yearly in a dental office. Justification is the most critical and fundamental paradigm of radiation protection. The safety report on radiation protection in dental radiology published by the International Atomic Energy Agency IAEA in 2022 points out that “justification for a CBCT examination has to follow scientifically generated evidence” [3]. CBCT scans must be acquired only according to the guidelines of the specific field of dentistry [3]. In children and juveniles, justification must be considered even more critical since the stochastic risk of radiation-induced carcinogenic damage in tissue is inversely correlated with age [8, 10]. However, we could not evaluate the justification behind the CBCTs in our cohort since these data could not be safely retrieved from the patient records over the entire five-and-a-half-year period. The reason is that these data were recorded electronically only since 2021. Furthermore, the justification is not stored in the i-Dixel databases searched by the algorithm. Safe retrieval of the justification with a search algorithm was technically not possible. Consequently, we would not have been able to retrieve the indication for most of the > 10,000 CBCT scans evaluated in the study. While including this information would have been very interesting, the large number of scans made a manual search over the mostly hand-written records almost impossible. An evaluation of the indication would only have been feasible for a small yet not representative subset. Hence, we decided to use the objective data available for all CBCTs over this period, providing valuable information on dose and radiation protection aspects. As we are currently implementing a completely digital hospital information system, it may also be possible to evaluate justification in a future study.

In light of this downside of the present study, our main goal was to look at the number of scans in the vulnerable group of young patients in combination with the respective exposure parameters for these CBCTs. The evaluation of more than 10,000 CBCTs showed that radiation protection aspects were only partly considered and that there is significant potential for improvement. Possible approaches are general dose reduction techniques such as the reduction of mA, kV, and exposure angles [14, 15].

Only a few studies with similar objectives can be found in the literature. Most of the published survey studies focus on the practice of CBCT use among dentists [16–18]. For Switzerland, guidelines for the use of CBCT have been published in 2014 [19]. Regarding CBCT exposures in children and juveniles, the guidelines postulate that “the usefulness of CBCT in pediatric dentistry is therefore limited to specific diseases of children. These are generally diseases with greater importance to the overall health of the child, such as specific or severe inflammations, bone diseases, benign and malignant tumors or other very special pathological conditions” [19]. We observed that roughly 20% of our CBCTs were acquired in patients under 25 years, yet with an increasing trend over the period from 2017 to 2022 (Table 1). Within this group, the percentage of patients under 18 years steadily increased from 7.2% in 2017 to 11.6% in 2022. This indicates an alarming trend from a radiation protection perspective. Particularly outcome quality of CBCTs acquired in such young patients will likely be compromised owing to their disability to remain steady over the scan time of several seconds. Spin-Neto and colleagues observed motion in 60 to 100% of patients up to 14 years of age in a realistic CBCT-simulation study [20]. For patients younger than 12 years, the odds ratio for movement increased to 2.41 with an upper 95% confidence interval bound of 4.70 [20]. It is a well-established fact that dental radiography is unique in terms of its high frequency of use in pediatric patients, including infants, children, and adolescents [3]. Nevertheless, a percentage of around 20% in the young age group under 25 years exposed to CBCT scans seems remarkable. Unfortunately, there is hardly any numeric information on the proportion of such dental radiographs in young patients available. In a retrospective study of three United Kingdom Dental Hospitals, Hidalgo and coworkers observed 13.7% of patients under 18 years undergoing CBCT imaging [21]. This is even more than in our sample (9.2%).

As FOV size significantly determines the effective patient dose [8, 22], we also looked at the distribution of FOV sizes. The latter were classified into “small”, “medium”, and “large” using a simple classification according to FOV height. This classification follows a conclusion from Ludlow et al. [8], who found that volume height when using DAP was a more accurate predictor of effective dose than beam area (FOV area) [8]. In our study, mainly containing data from the 3D Accuitomo 170 device (J. Morita Corp., Kyoto, Japan), effective doses of certain FOVs can be found in the literature (see e.g., Ludlow et al. [8]). For instance, the effective dose for a 4 × 4 cm volume range between 32 µSv and 43 µSv [23, 24], whereas for a 5 × 10 cm (height × width) FOV, the value range between 58 µSv and 297 µSv [8]. These findings show that even for a CBCT, we classified to “small” FOV (5 × 10 cm), relatively high effective doses can be accumulated. Considering the large percentage of juveniles among the patients in our study, it is crucial to realize that effective doses are higher in children than in mature patients when using identical exposure parameters [25]. This can be attributed to the fact that relatively more tissue is in the primary beam in this young patient group. The well-known ALARA (as low as reasonably achievable) principle postulates that the dose should be optimized to a level sufficient to answer the diagnostic question. Being slightly more instructive, recently ALADA (as low as diagnostically acceptable) has been postulated as a new acronym [26]. Moreover, the DIMITRA consortium proposed expanding the acronym to ALADAIP (as low as diagnostically acceptable being indication-oriented and patient-specific) [27]. Regardless of the acronym, our data indicate that despite these established concepts, it seems challenging to transfer them to the everyday clinical situation without even considering the justification.

If we look at the exposure parameters kV and mA, we notice that in many cases, the standard settings of CBCT for adult patients (90 kV, 5 mA) were applied. We also observed that the tube current (mA) was not regularly adapted to age in this cohort, indicating that additional dose-determining factors were often neglected. Several studies describe an excellent potential for dose reduction by mA with a minimal image quality loss [28, 29]. A concrete suggestion for the adjustment of mA values for the 3D Accuitomo 170 has been made by Pauwels et al. [14]. Ideally, mA is adjusted according to the circumference of the head. For ease of use, this can be assigned to age via average values and is gender specific. A possible suggestion on mA adaption according to age is as follows: 5 mA for males ≥ 17 years, 4.5 mA for males ≥ 13.5 years and females ≥ 18.5 years, 4 mA for males ≥ 9.5 years and females ≥ 12 years, 3.5 mA for males ≥ 5.25 years and females ≥ 7 years, 2.5 mA for males ≥ 2.5 years and females ≥ 3.25 years [14]. Therefore, for several months, the exposure parameters for children up to 18 years old for CBCT acquisition in our department are set to 80 kV, 3 mA, and a scan angle of 180°.

From a radiation protection perspective, it can be considered positive that 74.8% of CBCT scans were acquired with a small FOV in our cohort. It indicates that the basic paradigm of optimization was followed in our cohort to limit the FOV dimensions. FOV limitation is a basic paradigm for optimization in children and adolescents following the best clinical practice guidance for dental radiographs by the European Academy of Paediatric Dentistry (EAPD) [30].

DAP values should be compared to diagnostic reference levels (DRLs). DRL values help optimize radiation protection and serve as a reference of whether the patient dose is unusually high or low for medical imaging. In Switzerland, DRL values have been available since 2020 for CBCTs in the head and neck region [31]. The DRL values are given for five different indications of a CBCT with a FOV dimension of 5 × 5 cm. For four indications (wisdom tooth, single implant, form and position anomalies of tooth, dentoalveolar pathologies), DRL values are 450 mGycm^2^, and for endodontics, 640 mGycm^2^ [7, 31]. In the present sample, the mean DAP values in the age group up to 10 years were 331.1 mGycm^2^, and in the age group 10 to 20 years, 424.4 mGycm^2^. Thus, the DAP values of our sample were below the reference values. In individual cases, the values were significantly higher, for example, if a larger FOV was prepared. However, when comparing the DAP values in our cohort to a recent study conducted by Hung et al. on patient doses received during CBCT scans, we observe differences in the median values of the study populations [32]. Hung et al. found a median DAP of 333 mGycm^2^ for small FOV (≤ 40 cm^2^), which is lower than the DAP values in our cohort [32]. It is important to note that these values are difficult to compare due to the use of other CBCT devices in the respective studies.

Considering that many of the CBCTs at the dental clinic of the University of Bern are acquired for external referring offices, it seems obvious that education plays a significant role. It highlights the need for optimization of both undergraduate as well as postgraduate education on radiation risks and protection. In Switzerland and in the European Union, it is mandatory to take part in postgraduate courses on radiation protection in a 5-year frequency. Emphasizing the particular vulnerability of children and juveniles should be an important topic of such courses, especially in light of an ever-increasing number of CBCT scans. In addition, undergraduate education should aim for a fundamental awareness of all dental students concerning this topic.

Our retrospective observational evaluation has some limitations. It is certainly not representative of other similar university dental clinics. It is rather exemplary. Site-specific criteria have not been evaluated. Also, no referral reasons or general justification criteria have been included due to documentation shortcomings over the majority of the time period. A proper justification cannot be concluded from this study. This may be included in a future study at our division. Nevertheless, in this context, it should be noted that the sheer sample size combined with the university dental school environment and many additional external referrals from dental offices will surely provide a broad basis for different indications. Without looking into the specific indications, we can safely conclude on exposure parameters used for different age groups. These data are clearly indicative of general exposure measures and, thus, radiation protection in a clinical dental environment. The FOV size was classified based on the FOV height following the findings of Ludlow et al. [8]. The exact classification into the three categories (small, medium, large) is simple yet debatable. Another classification would obviously modify the outcome in parts, but not this study’s general observations. Despite these shortcomings, the large sample size and the relatively long observation time ensure an accurate overview of the general patient cohort undergoing CBCT imaging at our university dental clinic.

In conclusion, our retrospective study on the complete cohort of patients undergoing CBCT scans for various purposes in a central European university dental clinic demonstrates that radiation protection with reduction of the exposure parameters FOV, mA, kV, and scan angle was only partly considered. The study emphasizes the need to fully establish well-known radiation protection aspects in a regular daily clinical setting.

## Data Availability

The datasets used and analyzed during the present study are available from the corresponding author upon reasonable request.
